# Trajectories of functional performance recovery after inpatient geriatric rehabilitation: an observational study

**DOI:** 10.5694/mja2.51138

**Published:** 2021-06-16

**Authors:** Cheng Hwee Soh, Esmee M Reijnierse, Camilla Tuttle, Celia Marston, Rose Goonan, Wen Kwang Lim, Andrea B Maier

**Affiliations:** ^1^ The University of Melbourne Melbourne VIC; ^2^ Royal Melbourne Hospital Melbourne VIC; ^3^ Vrije Universiteit Amsterdam Amsterdam The Netherlands; ^4^ National University of Singapore Singapore

**Keywords:** Activities of daily living, Aged

## Abstract

**Objective:**

To identify functional performance trajectories and the characteristics of people who receive inpatient geriatric rehabilitation after hospital admissions.

**Design, setting, participants:**

REStORing health of acutely unwell adulTs (RESORT) is an observational, prospective, longitudinal inception cohort study of consecutive patients admitted to geriatric rehabilitation wards at the Royal Melbourne Hospital. Recruitment commenced on 15 October 2017.

**Main outcome measures:**

Functional performance, assessed with the Activities of Daily Living (ADL) and Instrumental Activities of Daily Living (IADL) scales two weeks before acute hospitalisation, on admission to and discharge from geriatric rehabilitation, and three months after discharge from geriatric rehabilitation.

**Results:**

A total of 618 rehabilitation patients were included in our analysis. For each of the two scales, three distinct functional performance trajectories were identified by latent class growth modelling: poor at baseline and 3‐month follow‐up (remained poor: ADL, 6.6% of patients; IADL, 42%), good at baseline but poor recovery (deteriorated: ADL, 33%; IADL, 20%), and good at baseline and good recovery (recovered: ADL, 60%; IADL, 35%). Higher Clinical Frailty Scale (CFS) score (*v* recovered, per point: odds ratio [OR], 2.51; 95% CI, 1.64–3.84) and cognitive impairment (OR, 6.33; 95% CI, 2.09–19.1) were associated with greater likelihood of remaining poor in ADL, and also with deterioration (CFS score: OR, 1.76; 95% CI, 1.45–2.13; cognitive impairment: OR, 1.87; 95% CI, 1.24–2.82). Higher CFS score (OR, 1.64; 95% CI, 1.37–1.97) and cognitive impairment (OR, 3.60; 95% CI, 2.31–5.61) were associated with remaining poor in IADL, and higher CFS score was also associated with deterioration (OR, 1.63; 95% CI, 1.33–1.99).

**Conclusions:**

Based on ADL assessments, most people who underwent inpatient geriatric rehabilitation regained their baseline functional performance. As higher CFS score and cognitive impairment were associated with poorer functional recovery, assessing frailty and cognition at hospital admission could assist intervention and discharge planning.

**The known**: Trajectories of functional performance during their hospital stay and after their discharge differ between older patients.

**The new**: Three functional performance trajectories were identified for older patients who underwent rehabilitation in hospital after acute admissions: remained poor, deteriorated, and recovered. Cognitive impairment and greater frailty were each associated with greater likelihood of remaining poor or deteriorating than recovery of functional performance.

**The implications**: Our findings indicate the importance of assessing the cognition status and frailty of older patients admitted to hospital, as these factors influence the likelihood of improvement in functional performance during inpatient rehabilitation.

Functional performance is defined as the ability to perform daily tasks of independent living, such as bathing, dressing, getting into and out of bed, eating, using the toilet, and being mobile in and around the home.^1^ A decline in functional performance in people aged 65 years or more is associated with poor quality of life and greater risk of hospitalisation, both of which are risk factors for institutionalisation and death.^2^ After acute hospitalisations, one‐third of older people experience functional decline,^3^ and the aim of geriatric rehabilitation is to restore the functional performance of such people.^4^

Trajectories of functional performance differ between older patients both during their hospital stay and after discharge.^5^ Assessing functional performance at three or more time points can help clinicians optimise patients’ functional performance with interventions such as physiotherapy and occupational therapy.^6^ As many as five distinct functional trajectories have been identified for older inpatients, including persistent disability, improvement from baseline disability, recovery from new disability, no recovery from new disability, and no disability.^7^ Older patients with marked loss of physical function during an acute hospitalisation are often admitted to inpatient geriatric rehabilitation to restore functional performance.^8^ However, trajectories of functional performance for these people have not been explored in detail. Identifying functional trajectories could guide rehabilitation strategies and increase the efficacy of interventions during their hospital stays.^5^

In this study, we examined trajectories of functional performance for older people, from two weeks prior to an acute hospitalisation to three months after discharge from inpatient geriatric rehabilitation. We also examined clinical characteristics associated with poor functional performance.

## Methods

REStORing health of acutely unwell adulTs (RESORT) is an observational, prospective, longitudinal inception cohort study of patients admitted to geriatric rehabilitation wards at the Royal Melbourne Hospital. Recruitment commenced on 15 October 2017; 693 consecutive patients discharged by 30 August 2018 were recruited in wave 1. Patients admitted for palliative care were excluded from our study.

### Patient characteristics

Patients with complex and multiple medical and functional conditions were admitted to geriatric rehabilitation wards after discharge from acute hospitalisations. Baseline patient characteristics were assessed within 48 hours of admission to geriatric rehabilitation wards with a multidimensional, multidisciplinary comprehensive geriatric assessment.^9^ Multimorbidity was assessed with the Charlson Comorbidity Index^10^ and Cumulative Illness Rating Scale;^11^ frailty was assessed with the Clinical Frailty Scale (CFS; 1 = fit; 9 = extremely frail).^12^

Cognitive impairment, assessed by physicians, was defined as being present if identified with the Charlson Comorbidity Index or Cumulative Illness Rating Scale, if dementia or cognitive impairment was noted in the discharge summary, or if the patient had a standardised Mini‐Mental State Examination score below 24 points, a Montreal Cognitive Assessment score below 26 points, or a Rowland Universal Dementia Assessment Scale score below 23 points.

The patients’ self‐reported quality of life was assessed at admission to geriatric rehabilitation with the EuroQol EQ‐5D‐5L;^13^ anxiety and depressive symptoms were assessed with the Hospital Anxiety and Depression Scale.^14^

### Functional performance

We assessed functional performance with the Katz Index of Activities for Daily Living (ADL)^15^ and the Lawton and Brody Instrumental Activities of Daily Living (IADL) scales.^16^ The Katz ADL is a 6‐point scale (0 = dependent; 6 = independent) for assessing bathing, dressing, using the toilet, transferring into and out of bed, continence, and feeding. The IADL is an 8‐point scale (0 = dependent; 8 = independent) that assesses the ability to use the telephone, undertake laundry, shopping, transportation, and food preparation, and to manage finances, medications, and housekeeping.

An occupational therapist assessed the functional performance at admission to and on discharge from geriatric rehabilitation. In addition, functional performance two weeks prior to the acute hospitalisation was assessed on the basis of information provided by the patients and their carers. Three months after discharge, the patients were assessed by trained research assistants with the ADL and IADL via telephone.

### Statistical analyses

Patients who died during their hospital admissions or the three‐month follow‐up period were excluded from our analyses. Patients’ admission characteristics are summarised as descriptive statistics: continuous variables with normal distributions as means with standard deviations (SDs), and those with skewed distributions as medians with interquartile ranges (IQRs).

The ADL and IADL were each administered at four time points: two weeks prior to admission to hospital, on admission to and discharge from geriatric rehabilitation, and three months after discharge from geriatric rehabilitation. Distinct trajectories of functional performance were identified by latent class growth modelling (LCGM); the total number of distinct latent trajectories was identified using the forward approach,^17^ starting with a model with one trajectory and then adding one trajectory at a time, modelling trajectory shapes by adding linear and quadratic terms. The fitness of models was assessed after each step by applying two criteria: the Bayesian information criterion, with a reduction of ten points or more defined as indicating improved model fit, and the mean posterior probabilities for patients in each trajectory group, with values exceeding 0.80 deemed recommendations; patients were allocated to the best fitting trajectory according to the largest posterior probability. The LCGM analysis was conducted in R (R Foundation for Statistical Computing).

Patients’ characteristics, stratified by ADL and IADL trajectories, are reported as descriptive statistics. Variables with missing data were analysed if the data were randomly missing. This applied to the CFS, the Hospital Anxiety and Depression Scale, and quality of life scores, and these were handled by multiple imputation (sequential regression multivariate imputation).^18^ Estimates were pooled according to Rubin’s rules.^18^

Clinical characteristics associated with each ADL and IADL trajectory were identified by multinomial regression, and the relationships quantified as odds ratios (ORs) with 95% confidence intervals (CIs). Variables for which *P* < 0.20 in the univariable analyses (likelihood ratio test) were included in the multivariable model. Descriptive statistics generation and regression analyses were conducted in SPSS Advanced Statistics 25.0 (IBM).

### Consent and ethics approval

Written informed consent was provided by all patients or their nominated proxies. The study was approved by the Melbourne Health Human Research Ethics Committee (HREC/17/MH/103) and followed the guidelines of the Declaration of Helsinki and the National Statement on Ethical Conduct in Human Research.

## Results

Of the 693 rehabilitation inpatients, 11 died during geriatric rehabilitation and 64 within three months of discharge from rehabilitation; 618 patients were therefore included in our analysis (online Supporting Information, figure). The patients’ median age was 83.1 years (IQR, 77.3–87.7 years) and 355 were women (57%); 479 patients had been admitted from acute medical wards (7.5%), 116 from surgical wards (19%) and 23 directly from home (3.7%). The most frequent primary reasons for hospital admission were musculoskeletal conditions (301 patients, 49%), neurological conditions (107, 17%), infections (74, 12%), cardiac conditions (46, 7.4%), and gastrointestinal conditions (42, 6.8%). The median Charlson Comorbidity Index score at admission was 2 (IQR, 1–4), the median Cumulative Illness Rating Scale score was 11 (IQR, 8–15); 396 patients (64%) had cognitive impairment. The median CFS score at admission was 6 (IQR, 5–6), and the median quality of life score 50 (IQR, 40–70). The median acute hospital length of stay was 7 days (IQR, 4–11 days), the median geriatric rehabilitation length of stay was 20 days (IQR, 13–29 days). Twenty patients (3.2%) had been institutionalised prior to hospital admission; three months after discharge from geriatric rehabilitation, 160 patients had been newly institutionalised (26%) (Box 1).

Box 1 **Patient characteristics on admission to geriatric rehabilitation**

**Table:** 

Characteristic	
Number of participants	618
Age (years), median (IQR)	83.1 (77.3–87.7)
Sex (women)	355 (57.4%)
Location prior to geriatric rehabilitation admission	
Acute medical wards	479 (77.5%)
Surgical wards	116 (18.8%)
Home	23 (3.7%)
Marital status (married)	242 (39.2%)
Living status (living alone)	247 (40.0%)
Primary reason for hospitalisation	
Musculoskeletal	301 (48.7%)
Neurological	107 (17.3%)
Infection	74 (12%)
Cardiac	46 (7.4%)
Gastrointestinal	42 (6.8%)
Other	48 (7.8%)
Charlson Comorbidity Index, median score (IQR)	2 (1–4)
Cumulative Illness Rating Scale, median score (IQR)	11 (8–15)
Cognitive impairment	396 (64.1%)
Hospital Anxiety and Depression Scale; Anxiety, median score (IQR)	5 (2–9)
Hospital Anxiety and Depression Scale; Depression, median score (IQR)	6 (3–10)
Clinical Frailty Scale, median score (IQR)	6 (5–6)
Quality of Life, median score (IQR)	50 (40–70)
Acute length of stay (days), median (IQR)	7 (4–11)
Geriatric rehabilitation length of stay (days), median (IQR)	20 (13–29)
Institutionalised prior to hospital admission	20 (3.2%)
New institutionalisation at 3‐month follow‐up	160 (25.9%)

IQR = interquartile range.

### Trajectories of functional performance

Forty‐one patients (6.6% of all patients) for whom ADL function was poor at both baseline and three months after discharge were classified as “remained poor”, 204 patients (33%) with good baseline ADL function but poor recovery were classified as “deteriorated”, and 373 patients (60%) with good baseline ADL function and good recovery were classified as “recovered”. Similarly, 262 patients (42%) were classified as “remained poor” for IADL function, 142 patients (23%) were classified as “deteriorated”, and 216 patients (35%) were classified as “recovered” (Box 2).

Box 2 **Activities of Daily Living (ADL) and Instrumental Activities of Daily Living (IADL) trajectories, from two weeks prior to acute hospital admission to three months after discharge from geriatric rehabilitation**

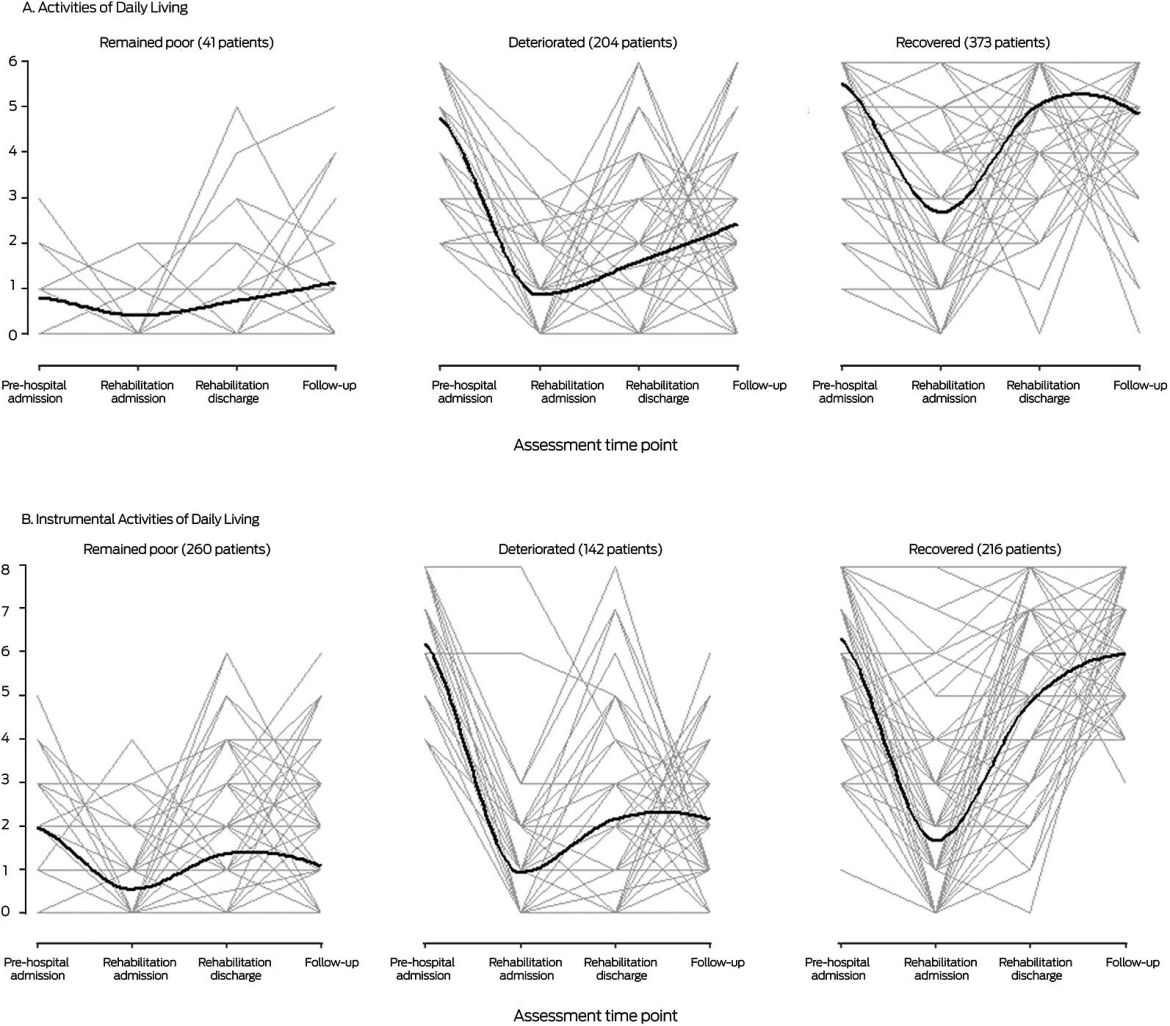

The median age and median frailty score were each highest for patients in the “remained poor” ADL and IADL trajectories, and the proportions with cognitive impairment were larger than for the “deteriorated” or “recovered” trajectories. By three months after discharge from rehabilitation, 13 patients who remained poor in ADL (32%) and 100 who remained poor in IADL (38%), and 95 patients who deteriorated in ADL (47%) and 49 who deteriorated in IADL (34%) had been newly institutionalised; 52 patients who recovered in ADL (14%) and eleven who recovered in IADL (5.1%) had been newly institutionalised (Box 3).

Box 3 **Patient characteristics, by Activities of Daily Living (ADL) and Instrumental Activities of Daily Living (IADL) trajectories**

**Table:** 

	ADL trajectories			IADL trajectories		
Characteristic	Remained poor	Deteriorated	Recovered	Remained poor	Deteriorated	Recovered
Number of participants	41	204	373	260	142	216
Age (years), median (IQR)	84.8 (80.0–87.3)	84.5 (78.5–88.2)	82.5 (76.1–87.3)	84.5 (78.7–88.3)	84.2 (78.6–88.1)	81.4 (75.3–85.9)
Sex (women)	17 (42%)	118 (58%)	220 (59%)	140 (54%)	75 (53%)	140 (65%)
Location prior to geriatric rehabilitation admission						
Acute medical wards	34 (83%)	158 (78%)	287 (77%)	216 (83%)	105 (74%)	158 (73%)
Surgical wards	5 (12%)	38 (19%)	73 (20%)	34 (13%)	30 (21%)	52 (24%)
Home	2 (5%)	8 (4%)	13 (4%)	10 (4%)	7 (5%)	6 (3%)
Marital status, married	28 (68%)	86 (42%)	128 (34%)	115 (44%)	54 (38%)	73 (34%)
Living status, living alone	4 (10%)	61 (30%)	182 (49%)	68 (26%)	57 (40%)	122 (56%)
Primary reason for hospitalisation						
Musculoskeletal	13 (32%)	92 (45%)	196 (52%)	113 (44%)	70 (49%)	118 (55%)
Neurological	14 (34%)	45 (22%)	48 (13%)	48 (18%)	34 (24%)	25 (12%)
Infection	5 (12%)	26 (13%)	43 (12%)	38 (15%)	13 (9%)	23 (11%)
Cardiac	1 (2%)	15 (7%)	30 (8%)	18 (7%)	12 (8%)	16 (7%)
Gastrointestinal	3 (7%)	11 (5%)	28 (8%)	18 (7%)	4 (3%)	20 (9%)
Other	5 (12%)	15 (7%)	28 (8%)	25 (10%)	9 (6%)	14 (6%)
Charlson Comorbidity Index, median score (IQR)	2 (1–4)	2 (1–4)	2 (1–4)	3 (1–4)	2 (1–4)	2 (1–3)
Cumulative Illness Rating Scale, median score (IQR)	12 (9–14)	12 (9–16)	11 (7–14)	12 (9–15)	11 (8–15)	11 (8–13)
Cognitive impairment	37 (90%)	149 (73%)	210 (56%)	208 (80%)	83 (58%)	105 (49%)
Hospital Anxiety and Depression Scale: anxiety, median score (IQR)	7 (2–12)	5 (2–10)	4 (1–8)	5 (2–10)	5 (2–9)	4 (1–9)
Hospital Anxiety and Depression Scale: depression, median score (IQR)	10 (6–13)	7 (3–12)	5 (2–9)	7 (3–11)	6 (3–9)	5 (2–8)
Clinical Frailty Scale, median score (IQR)	7 (6–7)	6 (6–7)	5 (4–6)	6 (5–7)	6 (5–7)	5 (4–6)
Quality of Life, median score (IQR)	33 (18–53)	50 (30–70)	60 (45–75)	50 (30–70)	50 (35–70)	60 (50–75)
Functional (ADL/IADL), median score (IQR)						
Two weeks before hospital admission	1 (0–1)	5 (4–6)	6 (5–6)	2 (1–3)	6 (5–7)	7 (5–8)
Admission to geriatric rehabilitation	0 (0–1)	1 (0–1)	2 (2–4)	0 (0–1)	1 (0–1)	1 (1–2)
Discharge from geriatric rehabilitation	0 (0–1)	1 (1–2)	5 (4–6)	1 (0–2)	2 (1–3)	5 (4–6)
Three months after discharge from geriatric rehabilitation	1 (0–1)	2 (1–3)	5 (4–6)	1 (0–2)	2 (1–3)	6 (5–7)
Acute length of stay (days), median (IQR)	6 (4–9)	7 (4–12)	6 (4–10)	6 (4–10)	7 (4–11)	7 (4–11)
Geriatric rehabilitation length of stay (days), median (IQR)	18 (15–28)	25 (16–35)	18 (12–26)	21 (13–30)	22 (15–35)	16 (12–25)
Institutionalised prior to hospital admission	3 (7%)	12 (6%)	5 (1%)	15 (6%)	3 (2%)	2 (1%)
New institutionalisation at 3‐month follow‐up	13 (32%)	95 (47%)	52 (14%)	100 (38%)	49 (34%)	11 (5%)

IQR = interquartile range.

All 41 patients who remained poor in ADL also remained poor in IADL; 131 patients who deteriorated in ADL (64%) remained poor, 63 deteriorated (31%), and ten recovered in IADL (5%). Among patients who recovered in ADL, 88 patients remained poor (24%), 79 deteriorated (21%), and 206 recovered in IADL (55%) (Box 4). Among patients with ADL trajectories of deterioration and recovery, larger proportions of patients with cognitive impairment remained poor in IADL (ADL, deteriorated, 108 of 131 [82%]; ADL recovered, 63 of 88 [72%]) than deteriorated or recovered (Supporting Information, table 1).

Box 4 **Instrumental Activities of Daily Living (IADL) trajectories, by Activities of Daily Living (ADL) trajectories**

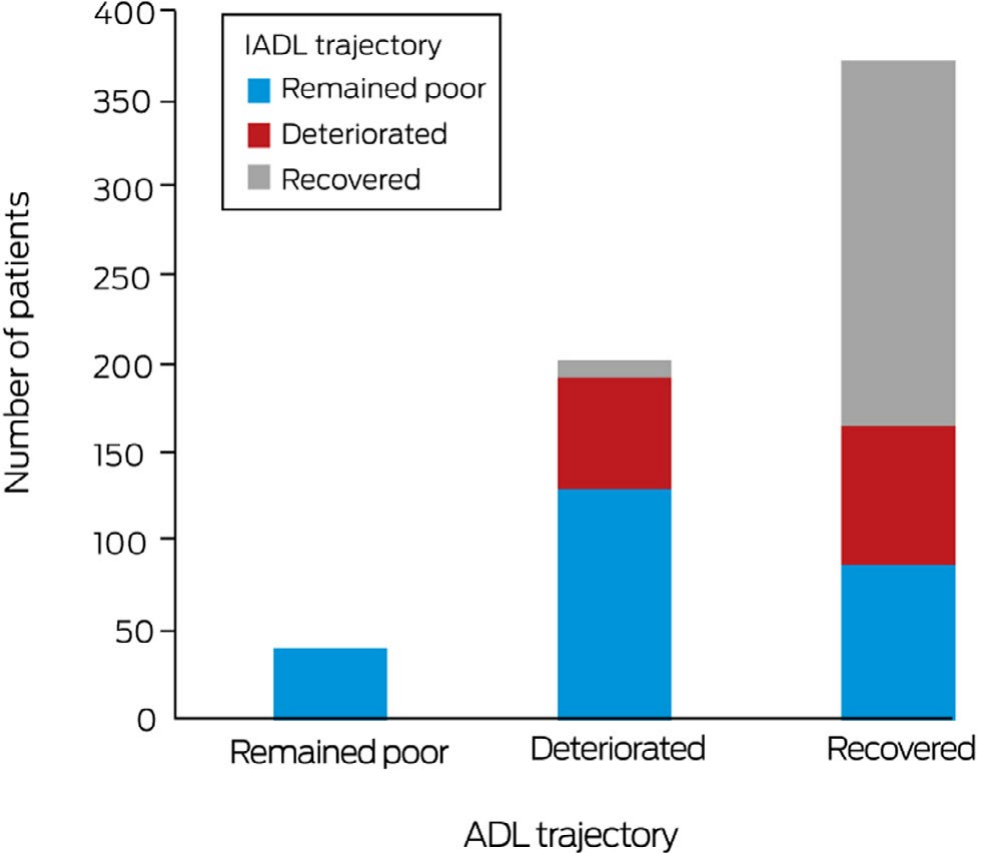

### Clinical characteristics associated with remaining poor or deteriorating in ADL

In univariable analyses, higher age, higher Cumulative Illness Rating Scale, CFS, and Hospital Anxiety and Depression Scale depression scores, cognitive impairment, and lower quality of life score were each associated with greater likelihood of deterioration than recovery; higher CFS score, being male, cognitive impairment, and lower quality of life score were also associated with greater likelihood of remaining poor (Supporting Information, table 2). In multivariable analyses, higher CFS score (*v* recovered, per point: OR, 2.51; 95% CI, 1.64–3.84) and cognitive impairment (OR, 6.33; 95% CI, 2.09–19.1) were associated with remaining poor, and also with deterioration (CFS score: OR, 1.76; 95% CI, 1.45–2.13; cognitive impairment: OR, 1.87; 95% CI, 1.24–2.82) (Box 5).

Box 5 **Characteristics that influence functional performance trajectories: multivariable analyses: odds ratios, with 95% confidence intervals, with recovery as reference trajectory**

**Table:** 

	Activities of Daily Living		Instrumental Activities of Daily Living	
Characteristic	Remained poor	Deteriorated	Remained poor	Deteriorated
Age, per year	1.00 (0.95–1.05)	1.01 (0.98–1.03)	1.02 (0.99–1.05)	1.03 (1.00–1.06)
Sex (women *v* men)	0.55 (0.27–1.13)	—	0.71 (0.47–1.08)	0.64 (0.40–1.01)
Charlson Comorbidity Index, per point	1.02 (0.86–1.21)	—	1.10 (1.00–1.23)	1.03 (0.93–1.15)
Cumulative Illness Rating Scale, per point	0.99 (0.91–1.09)	1.03 (0.99–1.07)	1.00 (0.95–1.05)	—
Cognitive impairment	6.33 (2.09–19.1)	1.87 (1.24–2.82)	3.60 (2.31–5.61)	1.22 (0.77–1.93)
Hospital Anxiety and Depression Scale: anxiety, per point	0.98 (0.88–1.10)	1.02 (0.96–1.09)	—	—
Hospital Anxiety and Depression Scale: depression, per point	1.06 (0.95–1.19)	1.03 (0.97–1.09)	1.04 (0.98–1.10)	1.03 (0.97–1.09)
Clinical Frailty Scale, per point	2.51 (1.64–3.84)	1.76 (1.45–2.13)	1.64 (1.37–1.97)	1.63 (1.33–1.99)
Quality of life, per point	0.98 (0.97–1.00)	1.00 (0.99–1.01)	0.99 (0.98–1.00)	—

### Clinical characteristics associated with remaining poor or deteriorating in IADL

In univariable analyses, higher age, being male, higher Charlson Comorbidity Index, Cumulative Illness Rating Scale, CFS, and Hospital Anxiety and Depression Scale depression scores, cognitive impairment, and lower quality of life score were each associated with greater likelihood of remaining poor than recovering; higher age, being male, and higher Charlson Comorbidity Index and CFS scores were also associated with greater likelihood of deteriorating (Supporting Information, table 2). In multivariable analyses, higher CFS score (*v* recovered, per point: OR, 1.64; 95% CI, 1.37–1.97) and cognitive impairment (OR, 3.60; 95% CI, 2.31–5.61) were associated with remaining poor, and higher CFS score was also associated with deterioration (OR, 1.63; 95% CI, 1.33–1.99) (Box 5).

## Discussion

We identified three distinct trajectories of ADL and IADL functional performance: patients for whom performance remained poor, deteriorated, or recovered. Cognitive impairment and higher CFS score at admission to geriatric rehabilitation were each associated with remaining poor and deteriorating on the two scales.

For two in three patients in the RESORT study, ADL functional performance had recovered by three months after discharge from geriatric rehabilitation, but IADL score had recovered in fewer than one‐half of the participants. An earlier study of older rehabilitation inpatients found that improvement between admission and discharge was slight, moderate or great for 89% of patients when assessed with the ADL, and for 73% when assessed with the IADL.^19^ These results indicate that a decline in ADL score during hospitalisation can be largely reversed, whereas recovery of IADL performance, which encompasses more complex daily tasks, is more difficult.^20^ Identifying patients at risk of poor functional trajectories could lead to personalised health care choices, including interventions for specific groups of rehabilitation inpatients.

Cognitive impairment was associated with poorer recovery of functional performance. Functional performance is inherently linked with a person’s cognitive abilities, including attention, executive, and visuospatial functioning, key determinants in diagnosing dementia.^1^ The association is also consistent with the reported effect of cognitive impairment on the functional decline of older rehabilitation patients during hospitalisation.^21^ A patient’s cognitive status and its impact on functional performance should be formally assessed during admission to hospital, both to assist with discharge planning, and to identify the support and services they may require after discharge.

Frailty is the state of increased vulnerability to poor restitution of homeostasis after stress, including that associated with hospitalisation.^12^ That higher CFS scores were associated with poor functional performance three months after discharge from rehabilitation is consistent with the finding of an earlier study that assessing frailty at hospital admission was useful for predicting functional outcome at discharge.^22^ The CFS is easy to use in clinical practice and can help clinicians choose individualised interventions.^23^

### Strengths and limitations

We have reported the first study to investigate functional performance trajectories from two weeks prior to acute hospitalisation to three months after discharge from inpatient geriatric rehabilitation. Previous studies have examined functional trajectories only in older patients with specific health conditions.^24^ Our findings indicate the clinical value of geriatric rehabilitation for identifying older patients at risk of no or poor recovery of functional performance, and could guide clinical interventions and discharge planning, providing better support and services after discharge. Moreover, RESORT is a large observational, inception cohort study in geriatric rehabilitation with few exclusion criteria, maximising the generalisability of its findings to other older people undergoing post‐hospitalisation rehabilitation.

In our study, however, functional performance prior to acute hospital admission and three months after discharge from inpatient rehabilitation was self‐reported, while it was directly assessed by occupational therapists on admission to and discharge from rehabilitation. Further, despite providing a sensitive and data‐driven method for classifying patients by trajectory, latent class growth modelling is influenced by variation in the analysed sample and therefore yields results that are less comparable with those of other studies than findings based on pre‐defined assessment cut‐off points.^25^

### Conclusion

While the functional performance of most patients in the RESORT cohort, as measured with the ADL, had recovered three months after discharge from geriatric rehabilitation, a considerable proportion of rehabilitation inpatients did not regain baseline levels of functional performance. Cognitive impairment and higher CFS scores were associated with functional performance remaining poor or deteriorating, indicating that assessing cognition and frailty at admission is important, and could assist with the design of rehabilitation interventions and discharge planning, optimising outcomes for patients.

## Competing interests

No relevant disclosures.

## Supporting information

Supplementary Material Supplementary methods and results
