# Supplementary material for: Trajectories of functional performance recovery after inpatient geriatric rehabilitation: an observational study
Source: Med J Aust. 2021 Jun 16;215(4):173–9. doi: 10.5694/mja2.51138 (PMC8453869; doi:10.5694/mja2.51138)
Supplement: Supplementary file 1 — Supplementary Material Supplementary methods and results [file MJA2-215-173-s001.pdf]

The Medical Journal of Australia

# Supporting Information

## Supplementary methods and results

**This appendix was part of the submitted manuscript and has been peer reviewed. It is posted as supplied by the authors.**

Appendix to: Soh CH, Reijnierse EM, Tuttle C, et al. Trajectories of functional performance recovery after inpatient geriatric rehabilitation: an observational study. *Med J Aust* 2021; doi: 10.5694/mja2.51138.

**Figure 1. Flow diagram for selection of participating patients**

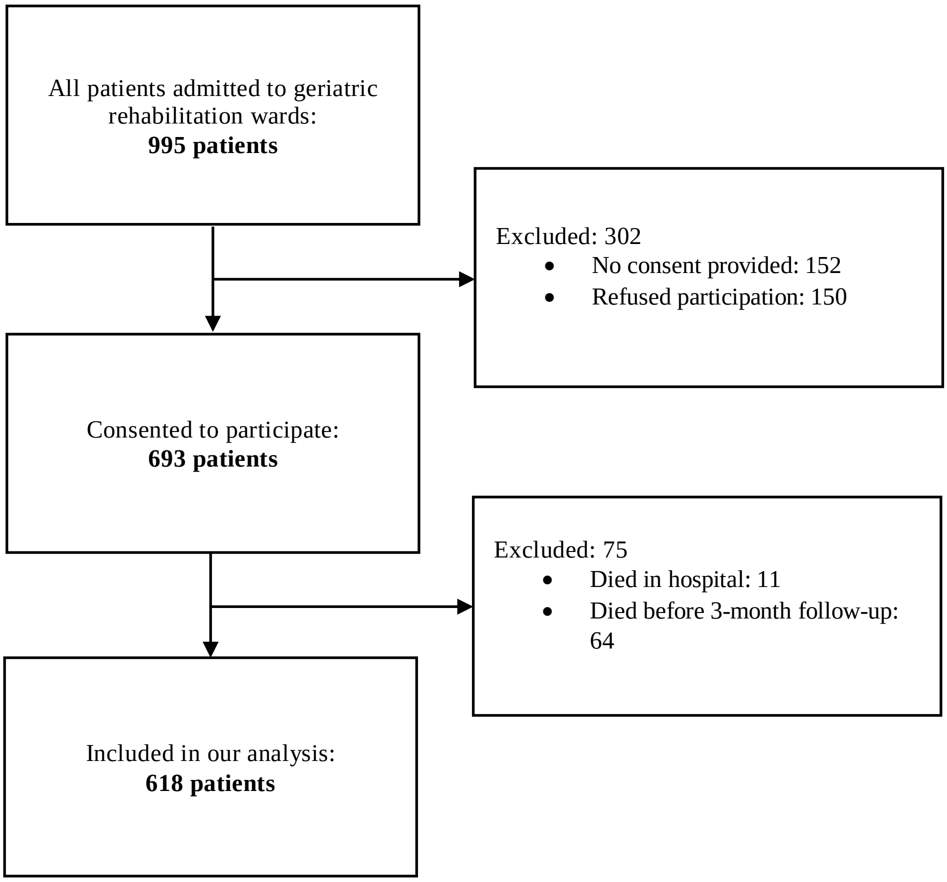

**Table 1. Patients' characteristics by Activities of Daily Living (ADL) (deterioration and recovery) and Instrumental Activities of Daily Living (IADL) trajectory**

| | ADL deteriorated | | | ADL recovered | | |
|---|---|---|---|---|---|---|
| | IADL: remained poor | IADL: deteriorated | IADL: recovered | IADL: remained poor | IADL: deteriorated | IADL: recovered |
| Number of participants | 131 | 63 | 10 | 88 | 79 | 206 |
| Age (years), median (IQR) | 84.7 (79.1–88.4) | 84.2 (77.9–87.6) | 82.1 (71.3–86.3) | 83.5 (75.9–89.3) | 84.4 (79.8–88.6) | 81.4 (75.4–86.0) |
| Sex (women) | 77 (59%) | 33 (52%) | 8 (80%) | 46 (52%) | 42 (53%) | 132 (64%) |
| Charlson Comorbidity Index, median score (IQR) | 3 (1–4) | 2 (1–3) | 4 (3–5) | 3 (1–5) | 2 (1–4) | 2 [1-3] |
| Cumulative Illness Rating Scale, median score (IQR) | 12 (9–17) | 10 (8–13) | 19 (12–22) | 12 (7–15) | 12 (9–15) | 10 (7–13) |
| Cognitive impairment | 108 (82%) | 36 (57%) | 5 (50%) | 63 (72%) | 47 (60%) | 100 (48%) |
| Hospital Anxiety and Depression Scale: anxiety, median score (IQR) | 5 (2–10) | 5 (3–10) | 10 (3–13) | 4 (1–9) | 6 (2–8) | 4 (1–8) |
| Hospital Anxiety and Depression Scale: depression, median score (IQR) | 7 (4–12) | 6 (3–10) | 10 (6–11) | 6 (3–10) | 6 (3–9) | 5 (2–8) |
| Clinical Frailty Scale, median score (IQR) | 6 (6–7) | 6 (5–7) | 7 (6–7) | 6 (5–6) | 6 (5–6) | 5 (4–6) |
| Quality of Life, median score (IQR) | 50 (30–70) | 50 (30–74) | 50 (25–70) | 60 (40–75) | 50 (37–70) | 60 (50–75) |
| Acute length of stay (days), median (IQR) | 7 (4–11) | 9 (6–13) | 7 (3–19) | 6 (3–10) | 6 (4–10) | 7 (4–11) |
| Geriatric rehabilitation length of stay (days), median (IQR) | 23 (15–33) | 29 (18–41) | 25 (16–38) | 18 (11–29) | 21 (13–28) | 16 (12–24) |
| Institutionalised prior to hospital admission | 11 (8%) | 1 (2%) | 1 (10%) | 1 (1%) | 2 (2%) | 2 (1%) |
| New institutionalisation at 3-month follow-up | 59 (45%) | 34 (54%) | 1 (10%) | 28 (32%) | 15 (19.) | 9 (4%) |

IQR = interquartile range.

**Table 2. Characteristics that influence functional performance trajectories: univariable analyses, with recovery as reference trajecto ry**

| | Activities of Daily Living | | Instrumental Activities of Daily Living | |
|---|---|---|---|---|
| | Remained poor | Deteriorated | Remained poor | Deteriorated |
| | OR (95% CI) | OR (95% CI) | OR (95% CI) | OR (95% CI) |
| Age, per year | 1.03 (0.99–1.08) | 1.02 (1.00–1.05) | 1.04 (1.02–1.06) | 1.04 (1.01–1.07) |
| Sex (women *v* men) | 0.49 (0.26–0.95) | 0.95 (0.68–1.35) | 0.63 (0.44–0.92) | 0.61 (0.40–0.94) |
| Charlson Comorbidity Index, per point | 1.10 (0.97–1.25) | 1.05 (0.97–1.13) | 1.16 (1.07–1.26) | 1.10 (1.00–1.22) |
| Cumulative Illness Rating Scale, per point | 1.05 (0.98–1.13) | 1.06 (1.02–1.10) | 1.06 (1.02–1.10) | 1.03 (0.98–1.08) |
| Cognitive impairment | 7.18 (2.51–20.6) | 2.10 (1.45–3.05) | 4.23 (2.82–6.34) | 1.49 (0.97–2.28) |
| Hospital Anxiety and Depression Scale: anxiety, per point | 1.05 (0.98–1.13) | 1.05 (1.00–1.09) | 1.02 (0.98–1.07) | 1.02 (0.97–1.07) |
| Hospital Anxiety and Depression Scale: depression, per point | 1.12 (1.03–1.21) | 1.07 (1.03–1.11) | 1.08 (1.03–1.13) | 1.04 (0.98–1.09) |
| Clinical Frailty Scale, per point | 2.55 (1.74–3.73) | 1.85 (1.54–2.21) | 1.79 (1.51–2.12) | 1.72 (1.41–2.09) |
| Quality of life, per point | 0.98 (0.96–0.99) | 0.99 (0.98–1.00) | 0.99 (0.98–0.999) | 0.99 (0.98–1.00) |

CI = confidence interval; OR = odds ratio.
